# Copaiba Oil-Resin Treatment Is Neuroprotective and Reduces Neutrophil Recruitment and Microglia Activation after Motor Cortex Excitotoxic Injury

**DOI:** 10.1155/2012/918174

**Published:** 2012-02-19

**Authors:** Adriano Guimarães-Santos, Diego Siqueira Santos, Ijair Rogério Santos, Rafael Rodrigues Lima, Antonio Pereira, Lucinewton Silva de Moura, Raul Nunes Carvalho, Osmar Lameira, Walace Gomes-Leal

**Affiliations:** ^1^Laboratory of Experimental Neuroprotection and Neuroregeneration, Institute of Biological Sciences, Federal University of Pará, 66075-900 Belém, PA, Brazil; ^2^Brain Institute, Federal University of Rio Grande do Norte, Natal, RN, Brazil; ^3^Laboratory of Ore Processing, Federal University of Pará, Marabá, PA, Brazil; ^4^Chemistry Laboratory, Federal University of Pará, Abaetuba, PA, Brazil; ^5^Laboratory of Biotechnology, EMBRAPA, Belém, PA, Brazil

## Abstract

The oil-resin of *Copaifera reticulata* Ducke is used in the Brazilian folk medicine as an anti-inflammatory and healing agent. However, there are no investigations on the possible anti-inflammatory and neuroprotective roles of copaiba oil-resin (COR) after neural disorders. We have investigated the anti-inflammatory and neuroprotective effects of COR following an acute damage to the motor cortex of adult rats. Animals were injected with the neurotoxin N-Methyl-D-Aspartate (NMDA) (*n* = 10) and treated with a single dose of COR (400 mg/kg, i.p.) soon after surgery (Group 1) or with two daily doses (200 mg/kg, i.p.) during 3 days (Group 2) alter injury. Control animals were treated with vehicle only. COR treatment induced tissue preservation and decreased the recruitment of neutrophils and microglial activation in the injury site compared to vehicle animals. The results suggest that COR treatment induces neuroprotection by modulating inflammatory response following an acute damage to the central nervous system.

## 1. Introduction

Inflammation is involved in several diseases of both neural and nonneural tissues [1]. After acute and chronic neural disorders a conspicuous inflammatory response takes place involving both humoral (i.e., immunoglobulins, cytokines) and cellular components (neutrophils, lymphocytes, astrocytes, and microglia/macrophages) [2].

Following acute neural disorders, including stroke and brain and spinal cord trauma, an intense inflammatory reaction is elicited [2]. In these diseases, recruitment of neutrophils and lymphocytes occurs in an early phase [3, 4] followed by an intense microglia/macrophage activation in latter stages [4–6].

 It has been shown that both neutrophils [7–9] and activated macrophages/microglia [10] contribute to secondary damage following experimental spinal cord injury (SCI) and stroke, as well as in humans [11, 12]. These inflammatory cells may release lytic enzymes, reactive oxygen species, and proinflammatory factors, contributing to tissue damage [13]. Experimental blockage of neutrophil recruitment with an anti-P-selectin monoclonal antibody [14] or inhibition of microglial activation with the semisynthetic tetracycline minocycline [10] induces neuroprotection following acute neural disorders. These anti-inflammatory therapies are a promising approach for central nervous system (CNS) human diseases [15, 16].

The copaiba oil-resin (COR) is obtained by tapping the trunk of the trees from several *Copaifera* L. species (Leguminoseae). These oleoresins have been traditionally used as healing and anti-inflammatory agents in the Brazilian folk medicine. Nevertheless, in recent years, several studies suggested a broad spectrum of COR effects in nonneural tissues, including anti-inflammatory and antinociceptive [17–19], cytotoxic and anticancerous [20], antimicrobial [21], wound healing, and antiulcer [18] activities. However, few studies have investigated the effects of COR on the nervous system [17] and mainly describe a general effect of COR treatment on CNS-mediated behaviors like anxiety [17]. So far, there are no investigations on the COR effects following an acute damage to the CNS.

In this study, we investigated the effects of COR treatment on tissue preservation, neutrophil infiltration, and microglia/macrophage activation following an NMDA-induced excitotoxic damage to the motor cortex of adult rats.

## 2. Material and Methods

### 2.1. Experimental Animals

 Male adult Wistar rats were obtained from the Central Animal Facility of the Federal University of Pará. All animals were housed under standard conditions with food and water available *ad libitum*. All experimental procedures were carried out in accordance with EU Directive 2010/63/EU for animal experiments UK and animals (Scientific Procedures) Act 1986 and associated guidelines as well as with the Principles of laboratory animal care [22], under license from the Ethics Committee on Experimental Animals of the Federal University of Pará. All efforts were made to avoid animal suffering and distress.

### 2.2. The Experimental Model of Excitotoxic Injury

 In our previous studies, we have characterized the present model of NMDA-induced excitotoxic injury in both the brain [3] and the spinal cord [5, 23]. In short, the animals were deeply anesthetized with an intraperitoneal injection of a mixture of Ketamine Chlorhydrate (90 mg/kg) and Xylazine Chloridrate (10 mg/kg) and positioned in a stereotaxic apparatus. A thermal blanket was used to maintain body temperature within the physiological range with the help of a rectal thermometer. After craniotomy, 80 nmol of NMDA (Sigma-Aldrich, St. Louis, MO, USA) in 1 *μ*L of sterile saline were injected into the left motor cortex over a period of 2 min using a glass capillary micropipette (*n* = 8 per survival time). The pipette was left in place for 3 minutes before being slowly withdrawn. Control animals were injected with the same volume of sterile saline (*n* = 5 per survival time). We used the following stereotaxic coordinates for the injection (in millimeters relative to bregma): 2.3 mm lateral; 1.2 mm posterior; 0.5 mm below the pial surface. To allow the posterior identification of the injection site, a small amount of colanyl blue was added to the injected solutions. After surgery, animals were allowed to rest in their cages with water and food ad libitum until postlesion days 1 and 4.

### 2.3. Harvest and Chemical Composition of the Copaiba Oil-Resin

The investigated oleo-resin was obtained by researchers from the Brazilian Agricultural Research Corporation (EMBRAPA) following the International Guidelines suggested by the World Health Organization (WHO) [24]. The COR was obtained by artificial exudation, through holes made in the trunk of the *Copaifera reticulata* Ducke tree. Two holes were made in the studied tree close to the trunk center in two different points (about 1 m and 1.5 m from the ground) using a 2 cm in diameter and 45 cm long drill. After the oleoresin harvest, both holes were plugged with a 3/4 diameter and 10 cm long tube in order to allow normal accumulation of the oleoresins. *Copaifera* tree was located in the experimental campus of EMBRAPA, in the district of Belterra, State of Pará, Brazil. The *Copaifera* tree has exsiccate kept in the West Amazon EMBRAPA'S IAN Herbarium (Belém-Pará-Brazil) under number 183939.

 The chemical composition of the COR was obtained by chromatographic analysis according to protocol published elsewhere [25]. A GC-MS equipment, in INCOSE Finnigan Mat XL system, equipped with silica capillary column DB-5MS (30 cm × 0.25 mm, 0.25 m film thickness) was used with the following operating conditions: carrier gas: helium at linear velocity of 32 cm/s (measured at 100°C); type of injection: “splitless” (1 mL of a solution 2 : 1000 hexane); injector temperature and detector: 250°C temperature program: 60°C−240°C (3°C/min); MS: electron impact, 70 eV; source temperature of ions and connecting pieces: 180°C. The components were identified by comparing their mass spectra and retention indices (RIs) with those of standard substances, the existing system libraries, and with literature data [26]. The IR was obtained using the homologous n-alkanes.

 The quantification of components was obtained by GC, HP5890-II, equipped with flame ionization detector (FID) and coupled to a HP3396 integrator-II under the same operating conditions, except that the carrier gas was hydrogen. Using this procedure, it was possible to obtain COR samples with high purity. The chemical composition of COR used in this investigation is shown in Table 1.

### 2.4. Treatment with Oil-Resin of *Copaifera  reticulata* Duke

 The COR was diluted in 5% Tween in sterile saline. Animals were treated with a single dose of COR (400 mg/kg, i.p.) soon after surgery (Group 1, *n* = 8) or with two daily doses (200 mg/kg, i.p) for 3 days (Group 2, *n* = 8) postinjury. Control animals were treated with vehicle only (*n* = 5). Animals from Groups 1 and 2 were perfused at 1 and 4 days after NMDA injection, respectively. These survival times were chosen because in our previous studies we have shown that the maximum numbers of neutrophils and activated macrophage/microglia in the damaged CNS occur at 1 and 4–7 days following NMDA injection [3, 5, 23]. The COR dose was chosen based on previous studies, which have shown that COR treatment at a 400 mg/kg dose induces a conspicuous decrease on both neutrophilic infiltration and lipoperoxidation following acetic-acid-induced colitis in adult rats [18].

### 2.5. Perfusion and Histological Analysis

 After the specified survival times, animals were deeply anesthetized with an overdose of Ketamine/Xylazine and perfused transcardially with 0.9% heparinized saline followed by 4% paraformaldehyde in 0.1 M phosphate buffer (PB), pH 7.4. The brains were removed from the skull, washed in 0.1 M PB for 5 minutes, and cryoprotected by immersion in increasing concentrations of sucrose solution. Coronal sections were obtained using a cryostat (Carl Zeiss Microm, Germany). Sections were collected onto gelatinized slides over microtomy and air dried for 24 h. After, slides were stored at −20°C for posterior analysis.

### 2.6. Histopathological Analysis and Immunohistochemistry

 The lesion area was visualized in coronal sections (20 and 50 *μ*m thick) stained with cresyl violet. The site of NMDA injection was recognized by the presence of colanyl blue and through the tissue pallor associated with loss of cell bodies.

In order to analyze both neutrophil infiltration and microglial activation, we resorted to standard immunohistochemical procedures. Neutrophils were labeled using the polyclonal antibody MBS-1 (1 : 1000), which recognizes epitopes present on the majority of neutrophil populations (a kind gift of Professor Victor Hugh Perry, CNS Inflammation Group, University of Southampton, UK). Activated microglia/macrophages were labeled using the antibody ED1 (1 : 500, Serotec, UK), which binds to an epitope on the lysosomal membrane of activated macrophages and microglia [27].

### 2.7. Labeling Protocol

 The slide-mounted sections were removed from the freezer, kept in an oven at 37°C for 30 minutes and rinsed in 0.1 M phosphate buffer saline (PBS) for 5 min. To improve labeling intensity, sections were treated with 0.2 M boric acid (pH 9.0) previously heated to 65°C for 25 min. This temperature was maintained constant over the treatment period. Sections were allowed to cool down for about 20 min and incubated under constant agitation in an 1% hydrogen peroxide solution in methanol for 20 min. Sections were then rinsed in 0.05% PBS/Tween (Sigma Company, USA) solution for 5 min for three times and incubated with 10% normal goat (MBS-1) or horse (ED1) serum in PBS for 1 h. Without further rinsing, sections were then incubated overnight with the primary antibody in PBS, rinsed in PBS/Tween solution for 5 min (3 times), and incubated with biotinylated goat anti-rabbit (MBS-1 antibody) or horse anti-mouse (ED1 antibody) secondary antibodies (Vector Laboratories, USA) diluted at 1 : 200 or 1 : 500 in PBS, respectively, for 2 hs. As a negative control, normal sera, rather the primary antibody, were used in some sections. Sections were rinsed again for 5 min (three times) and incubated in the avidin-biotin-peroxidase complex (ABC Kit, Vector Laboratories, USA) for 2 hs. Sections were rinsed four times (5 min each) and revealed with DAB [23]. After the DAB reaction, sections were rinsed two times (5 min each) in 0.1 M PB, dehydrated, and coverslipped with Entellan (Merck, Germany). Some sections were counterstained with cresyl violet.

### 2.8. Qualitative and Quantitative Analyses

 All sections were initially surveyed by light microscopy. Illustrative images from all experimental groups were obtained with a digital camera (Nikon Coolpix 950E) attached to a microscope (Nikon AFX-DX Optiphot-2).

We used coronal sections containing the damaged motor cortex to establish the areal density of neutrophils and activated macrophages/microglia (MBS-1 and ED1+ cells/mm^2^) using a 0.0665 mm^2^ square graticule attached to the microscope eyepiece (objective 40x). We counted 3 fields per section and 3 sections/animal for all experimental groups. The regions of interest had the highest cell density along a line passing through the lesion center (central field), and 2 additional fields were chosen at 1 mm intervals (1 mm medially and 1 mm laterally).

We used the Student's *t*-test for independent samples for the comparison between experimental and control groups, with significance level set at *P* < 0.01. Average values were expressed as mean ± S.E.M. All statistical analyses were done using the software Biostat [28].

## 3. Results

### 3.1. Excitotoxic Damage Induced by NMDA Injection

**Figure 1:**
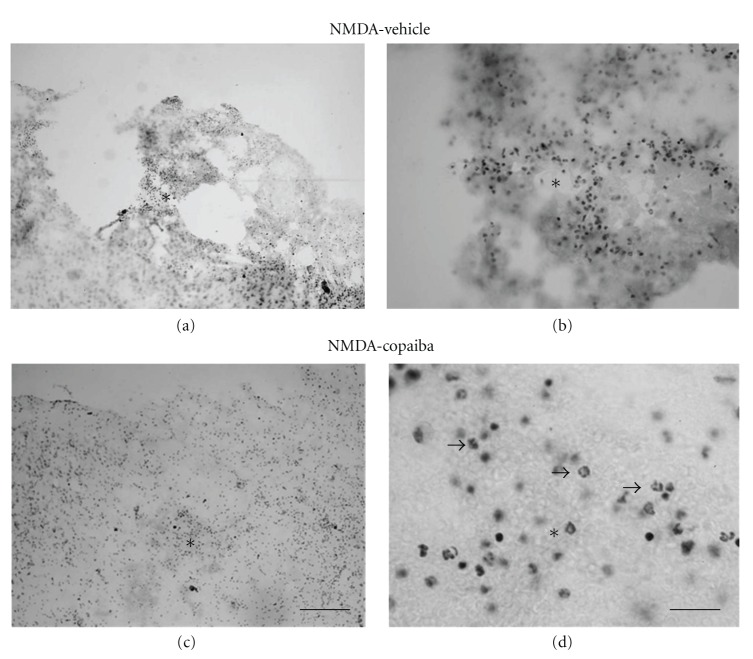
COR treatment effects on gross histopathology at 1 day following NMDA injection into the rat motor cortex, as revealed by cresyl violet staining. Animals treated with 5% tween (a-b) or 400 mg/kg of COR (c-d). COR treatment reduced polymorphonuclear cell infiltration and tissue loss (c-d) compared to vehicle animals (a-b). Arrows point to polymorphonuclear cells (d). Asterisks indicate the excitotoxic necrotic center. Scale bars: (a–c) (300 *μ*m); (b–d) (50 *μ*m).

**Figure 2:**
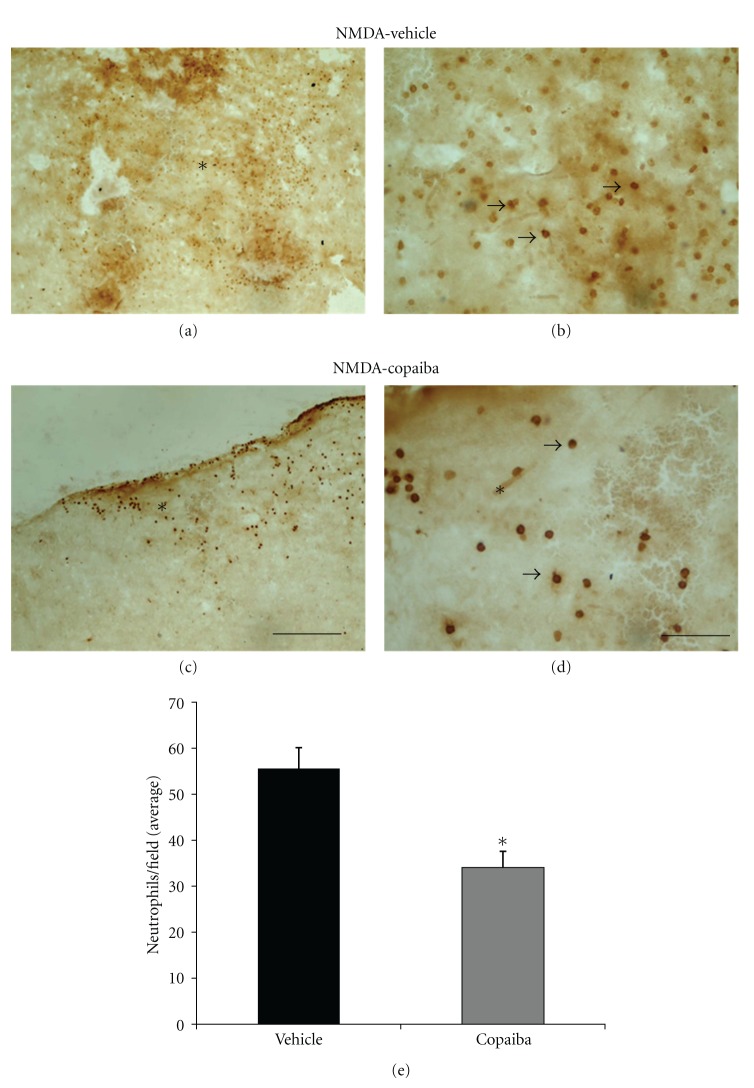
COR treatment reduces neutrophil infiltration at 1 day following cortical damage, as revealed by antineutrophil immunohistochemistry. Animals treated with 5% tween (a-b) or 400 mg/kg of COR (c-d). COR treatment reduced neutrophil infiltration (c-d) compared to vehicle animals (a-b), as confirmed by quantitative analysis (e, *P* < 0.01, Student's *t*-test). Arrows point to MBS-1+ cells (neutrophils). Asterisks indicate the excitotoxic necrotic center. Scale bars: (a–c) (300 *μ*m); (b–d) (50 *μ*m).

 Microinjections of 80 nmol of NMDA into the motor cortex of adult rats induced conspicuous tissue damage and inflammatory response at 1 day alter injection, as revealed by cresyl violet staining (Figures 1(a)-1(b)). Damage was characterized by tissue pallor, necrosis, and cavitation concomitant with intense infiltration of polymorphonuclear cells (Figures 1(a)-1(b)). The presence of neutrophils in the damaged tissue was confirmed by immunohistochemistry (Figures 2(a)-2(b)). Massive neutrophilic infiltration (MBS-1+ cells) was observed in the motor cortex at 1 day following NMDA injection, as confirmed by quantitative analysis (Figure 2(e)).

**Figure 3:**
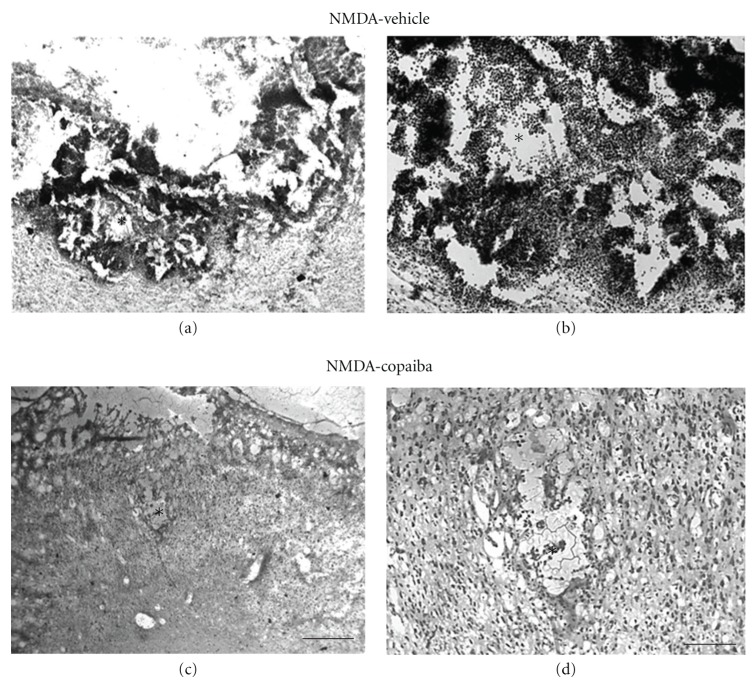
COR treatment effects on gross histopathology at 4 days following NMDA injection into the rat motor cortex, as revealed by cresyl violet staining. Animals treated with 5% tween (a-b) or 400 mg/kg of COR (c-d). COR treatment reduced mononuclear cell infiltration and tissue cavitation (c-d) compared to vehicle animals (a-b). Arrows point to mononuclear cells (d). Asterisks indicate the excitotoxic necrotic center. Scale bars: (a–c) (300 *μ*m); (b–d) (50 *μ*m).

**Figure 4:**
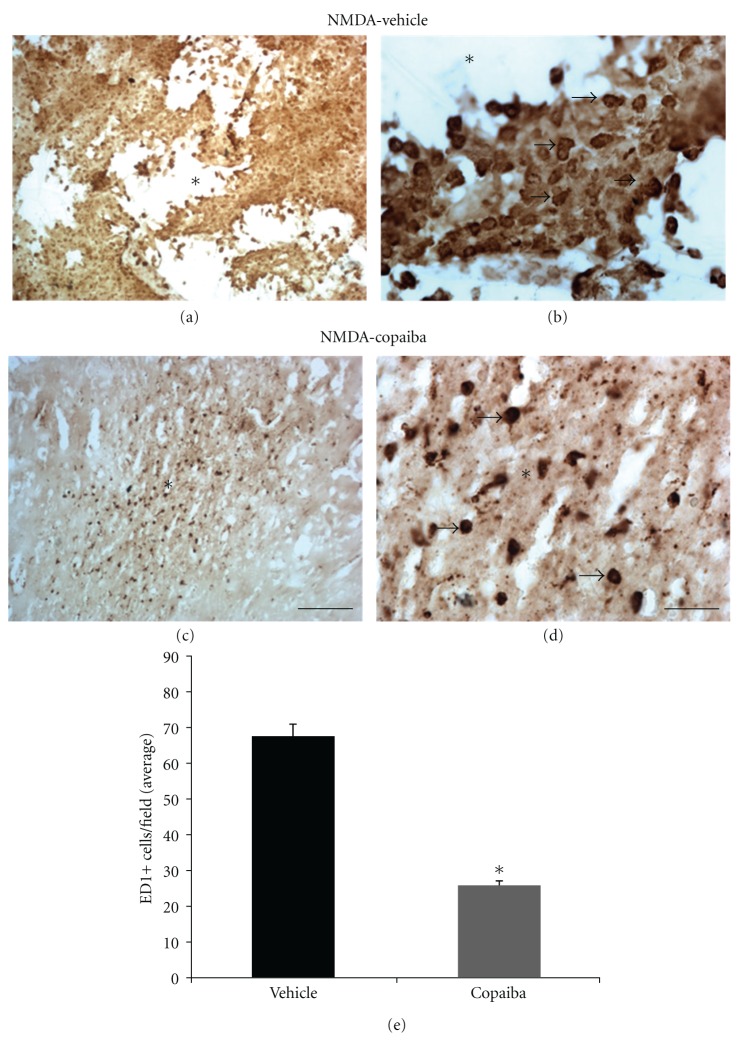
COR treatment reduces mononuclear cell infiltration at 4 days following cortical damage, as revealed by anti-ED1 immunohistochemistry. Animals treated with 5% tween (a-b) or 400 mg/kg of COR (c-d). COR treatment reduced microglia/macrophage activation (ED1+ cells) (c-d), compared to vehicle animals (a-b), as confirmed by quantitative analysis (e, *P* < 0.01, Student's *t*-test). Arrows point to ED1+ cells (activated microglia/macrophages). Asterisks indicate the excitotoxic necrotic center. Scale bars: (a–c) (300 *μ*m); (b–d) (50 *μ*m).

There was an increase in the necrotic area at 4 days after NMDA injection (Figures 3(a)-3(b)). Cavitations were more prominent in the damaged motor cortex (Figures 3(a)-3(b)). The inflammatory response was characterized by a dramatic increase in the number of mononuclear cells in and around the lesion site, as revealed by cresyl violet staining (Figures 3(a)-3(b)). Cell identity was confirmed by immunohistochemistry against ED1, a specific marker of activated microglia/macrophages (Figures 4(a)-4(b)), and confirmed by quantitative analysis (Figure 4(e)).

The neuropathological results described above are in agreement with previous studies by our own group [3, 23] and other authors [29] using the experimental model of excitotoxic damage using NMDA microinjections.

### 3.2. Copaiba Oil-Resin Treatment Induces Tissue Preservation and Reduces Neutrophil Recruitment and Microglia/Macrophage Activation following Acute Motor Cortex Damage

 In order to investigate the effect of COR treatment on tissue preservation and inflammatory response following acute motor cortex damage, we treated NMDA-injected animals with either COR or vehicle solution. There was less tissue damage in NMDA-injected animals treated with COR compared to vehicle animals both at 1 day (Figures 1(c)-1(d)) and 4 days alter injury (Figures 3(c)-3(d)). Tissue necrosis and cavitation were less evident in COR-treated animals (Figures 1(c)-1(d) and 3(c)-3(d)) compared to vehicle animals (Figures 1(a)-1(b) and 3(a)-3(b)).

 COR treatment also induced a conspicuous decrease in the number of both polymorphonuclear (Figures 1(c)-1(d) and 2(c)-2(d)) and mononuclear cells (Figures 3(c)-3(d) and 4(c)-4(d)) in the injured motor cortex, compared to vehicle animals ( Figures 1(a)-1(b) and 4(a)-4(b)). These results were confirmed by quantitative analysis (Figures 2(e) and 4(e), *P* < 0.01). The average numbers of neutrophils and activated microglia/field were 55.56 (±4.00) and 34.06 (±3.50) for animals treated with COR and the vehicle solution, respectively (Figure 2(e)). The corresponding numbers for activated microglia/macrophages were 75.59 (±3.00) and 28.70 (±2.14), respectively (Figure 4(e)). The data analysis shows that there was a 39% reduction of neutrophilic infiltration and a 62% decrease on microglial activation (Figure 4(e)).

## 4. Discussion

We investigated the effects of COR treatment on tissue preservation and inflammatory response following NMDA-induced excitotoxic damage to the motor cortex of adult rats. This experimental model of acute CNS damage is well established [3, 5, 30] and present neuropathological findings, including tissue necrosis and an intense inflammatory response. COR treatment reduced tissue necrosis and cavitation as well as neutrophil recruitment and microglia/macrophage activation in NMDA-injected animals, compared to the control group.

 COR has been traditionally used in Brazilian folk medicine as a healing and anti-inflammatory agent and recent investigations confirmed these effects in peripheral tissues both *in vitro* and *in vivo* [17–19]. Nevertheless, there are no published reports on the COR effects during acute damage to the CNS.

 A single intraperitoneal application of 400 mg/kg of COR from *Copaifera reticulata* Duke reduced damage to the motor cortex and neutrophil infiltration in about 39% at 1 day alter injury. This is in agreement with previous studies on peripheral tissues [17–19]. It has been shown that COR treatment (200 and 400 mg/kg) reduces neutrophilic infiltration and colonic mucosal damage following acetic-acid-induced colitis in rats [18]. In this study, COR treatment also reduced myeloperoxidase activity, further confirming the specific actions of COR treatment on neutrophil recruitment [18]. Anti-inflammatory effects of COR were also observed following experimental pleurisy [17] and skin flap reperfusion-ischemia [31] in rodents.

 Antineutrophilic effects of COR may partly explain the neuroprotection found in the present study. It has been shown that neutrophil recruitment is associated with damage exacerbation following experimental acute neural disorders, including SCI [9, 14] and stroke [8] as well as following human diseases [11, 32]. Inhibition of neutrophil elastases induces neuroprotection and improves functional recovery following rat spinal cord compression [9]. Moreover, inhibition of neutrophil recruitment by an anti-P-selectin monoclonal antibody renders similar results following SCI [14]. In addition, a recent investigation suggests that microglia may be beneficial by direct engulfment of both apoptotic and viable neutrophils following experimental ischemia [8].

 COR treatment reduced in more than 60% the levels of microglia/macrophage activation following acute cortical damage. This level of microglia/macrophage inhibition is superior to the one obtained by using minocycline, a classical microglia/macrophage inhibitor [10, 30]. This finding has also important neuroprotective implications, as it has been shown that overactivated macrophage/microglia contribute to tissue injury following CNS diseases [10, 16, 30], even in humans [15].

 Inhibition of microglial activation with minocycline induces a considerable decrease in both cortical and striatal infarct area following MCAO in rats [10]. Minocycline treatment protects oligodendrocytes, attenuates axonal degeneration, and improves functional outcome following SCI [33]. We have also shown that minocycline treatment protects the white matter following NMDA-induced striatal damage [30]. A recent study has shown that microglial activation is a long-lasting phenomenon following human stroke and may contribute to the physiopathology of this important disease [34].

 The mechanisms by which COR exerts its anti-inflammatory and neuroprotective effects are presently unknown. As discussed above, attenuation of neutrophil recruitment and inhibition of macroglia/macrophage activation may contribute to neuroprotection. These inflammatory cells may release oxygen radicals, proteases, and proinflammatory cytokines, like tumor necrosis factor alpha (TNF-*α*), contributing to tissue damage [35, 36]. COR might modulate the release of these proinflammatory substances, which would contribute to neuroprotection.

 COR chemical composition comprises several sesquiterpenes, including *β*-caryophyllene, *α*-copaene, and *α*-humulene, as revealed by chromatographic studies [17, 19, 25]. *β*-caryophyllene is the main sesquiterpene present in copaiba oil, comprising 40% to 57% of COR composition, depending on the *Copaifera* specie [19]. In *Copaifera reticulata *Ducke, the species used in the present study, *β*-caryophyllene comprises about 40% of COR composition [19], which is in agreement with our own chromatographic studies.

*β*-caryophyllene is a putative candidate as the main anti-inflammatory COR compound. *In vitro* studies suggest that *β*-caryophyllene is a dietary cannabinoid with important anti-inflammatory effects through the inhibition of cannabinoid type-2 (CB(2)) receptor [37]. *In vitro*, this plant sesquiterpene binds selectively to CB(2) receptor inhibiting proinflammatory pathways, including toll-like receptor complex CD14/TLR4/MD2, which normally leads to the expression of IL-1*β*, IL-6, IL-8, and TNF-*α* 9 [37]. *In vivo*, oral treatment with *β*-caryophyllene decreases inflammation in a mouse model of colitis [38]. It is possible that that a downstream mechanism of *β*-caryophyllene might be the decrease of TNF-*α* release by inhibition of CB-2 cannabinoid receptor [39]. In addition, other sesquiterpenes may be involved on the mechanisms underlying the COR anti-inflammatory effects. *α*-humulene, other sesquiterpene present in the COR, reduces eosinophil recruitment and release of several proinnflammatory substances in an experimental model of airways allergic inflammation [40] or following LPS injection in the rat paw [41]. It is possible that different sesquiterpenes interact synergistically contributing to the anti-inflammatory effects of COR observed in the present study.

## 5. Conclusions

In this study, we provide the first experimental evidence that COR is an anti-inflammatory and neuroprotective agent following an acute damage to the CNS. COR sesquiterpenes, including *β*-caryophyllene, are likely responsible for the anti-inflammatory and neuroprotective effects here described. This hypothesis should be investigated in further studies using molecular biology techniques in different *in vitro* and *in vivo* experimental models of CNS disorders. Considering that *β*-caryophyllene is already an FDA-approved food additive, these natural sesquiterpenes have an enormous potential to decrease the burden of inflammation-induced damage following neural and nonneural diseases.

## Figures and Tables

**Table 1:** Composition of the copaiba's oleoresin used in the present study revealed by gas chromatography.

Component	Percentage (%)
*δ*-elemene	0.2
cyclosativene	0.9
*α*-copaene	0.5
*δ*-elemene	0.2
cyclosativene	0.9
*α*-copaene	0.5
*β*-elemene	3.2
*α*-gurjunene	0.7
*β*-caryophyllene	37.3
*trans*-*α*-bergamotene	9.0
aromadendrene	0.9
*epi-β* *-*santalene	0.1
*α*-humulene + (E)-*β*-farnesene	5.4
*β*-chamigrene	1.0
*γ*-gurjunene	0.6
*γ*-curcumene	0.6
*β*-selinene	4.8
*α*-selinene	3.0
(Z)-*α*-bisabolene	1.8
*α*-bulnesene	2.2
*β*-bisabolene	14.5
*β*-curcumene	0.4
*β*-sesquiphelandrene	1.2
(E)-*γ*-bisabolene	1.4
caryophyllene oxide	0.1
*epi*-*β*-bisabolol	0.1
*β*-bisabolol	0.2
